# Assessing the Activation of Tyrosine Kinase KIT through Free Energy Calculations

**DOI:** 10.1021/acs.jctc.2c00526

**Published:** 2022-09-27

**Authors:** Angélica Sandoval-Pérez, Beth Apsel Winger, Matthew P. Jacobson

**Affiliations:** † Department of Pharmaceutical Chemistry, University of California, San Francisco, San Francisco 94158, California, United States; ‡ Department of Pediatrics, Division of Hematology and Oncology, University of California, San Francisco, San Francisco 94158, California, United States

## Abstract

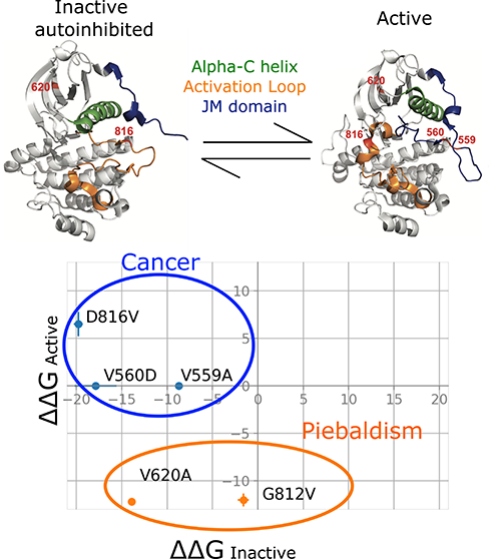

KIT is a type 3 receptor tyrosine kinase that plays a crucial role in cellular growth and proliferation. Mutations in KIT can dysregulate its active–inactive equilibrium. Activating mutations drive cancer growth, while deactivating mutations result in the loss of skin and hair pigmentation in a disease known as piebaldism. Here, we propose a method based on molecular dynamics and free energy calculations to predict the functional effect of KIT mutations. Our calculations may have important clinical implications by defining the functional significance of previously uncharacterized KIT mutations and guiding targeted therapy.

## 1 Introduction

KIT is a type 3 receptor tyrosine kinase that plays a critical role in cellular growth and proliferation.^1,2^ KIT is activated when its ligand, stem cell factor, binds to the extracellular domain, leading to homodimerization, trans-phosphorylation, and activation of the intracellular kinase domain. The juxtamembrane (JM) domain plays an important regulatory role in this activation process. In the inactive state, the JM domain adopts an autoinhibitory conformation, blocking the active site of the kinase.^3^ Once activated, the JM domain moves away from the active site, making the active site accessible to the substrate (Figure S1). Within the kinase domain, two other important conformational changes occur during KIT activation: (1) the activation loop reorients to permit peptide substrate binding and (2) the αC helix moves toward the active site where it can coordinate the other KIT substrate, adenosine triphosphate (ATP) (Figure S1).^4,5^ The fully activated kinase domain then transfers the gamma phosphate of ATP to protein substrates, leading to activation of cellular growth pathways, including the mitogen-activated protein kinase pathway and the phosphoinositide-3-kinase pathway.^6−8^

KIT plays an important role in the development of gametes, blood cells, mast cells, and melanocytes.^2^ Loss of function mutations cause the autosomal dominant condition, piebaldism, which is characterized by loss of pigmentation of the skin and hair.^9^ Gain of function mutations are found in several types of cancers, including gastrointestinal stromal tumors, acute myelogenous leukemia, systemic mastocytosis, and germinomas.^10−18^

Activation of wild-type (WT) KIT is tightly regulated, but oncogenic mutations dysregulate the protein, leading to overactivation and uncontrolled growth.^19^ These mutations most frequently occur in either the autoinhibitory JM domain or in the activation loop of the kinase domain.^10,20,21^ The structural mechanisms of activation are incompletely characterized, but mutations in the JM domain are thought to relieve autoinhibition, whereas mutations in the kinase domain are thought to cause constitutive activation.^7,19^ Understanding mechanisms of oncogenic KIT activation has been essential in the development of potent and specific KIT inhibitors. Several U.S. Food and Drug Administration-approved ATP-competitive small-molecule KIT kinase inhibitors have improved the outcome in KIT-driven cancers.^10,19,22^ However, these drugs vary in their ability to bind to oncogenic mutants. Drugs such as imatinib and regorafenib are effective against KIT JM domain mutations, for example, V559A and V560D, but ineffective against KIT activation loop mutations, such as D816V. Structural studies using X-ray crystallography have shown that regorafenib and imatinib only bind the inactive KIT conformation and therefore cannot bind to activation loop mutants that are constitutively active.^23^ In contrast, newer KIT inhibitors, such as avapritinib,^4^^24^ and midostaurin,^25,26^ bind to KIT in the active conformation and are therefore effective against both JM and activation loop mutants.

Efforts to develop more potent and specific KIT inhibitors continue^27,28^ and are guided by structural changes that occur during KIT activation.^4,29^ However, no crystal structure of a KIT D816V has been reported, and the mechanism of activation of activation loop mutations represents a current knowledge gap. Computational methods such as molecular dynamics (MD), meta-dynamics, and alchemical free energy calculations are valuable tools to explore the dynamic and the conformational landscape of kinases. We have previously used these methods to characterize the mechanism by which KIT mutations lead to kinase inhibitor resistance.^30−33^ In this work, we sought to use MD and alchemical free energy calculations to gain insights into the mechanism of activation of KIT mutants, in comparison to the WT. Our calculations predict that oncogenic mutations in the activation loop and JM domain in KIT destabilize the inactive conformation and that the activation loop mutant, D816V, additionally stabilizes the active conformation. Moreover, both of the piebald mutations destabilize the active conformation, and the mutation V620A additionally destabilizes the inactive form. Similar calculations could be applied to other kinase mutations to predict activation/deactivation through destabilization or stabilization of the inactive and active conformations.

## 2 Results

### 2.1 Oncogenic Mutations in the JM Domain and the Piebald Mutation in the Activation Loop Affect the Global Structure and Dynamics of KIT

**Figure 1:**
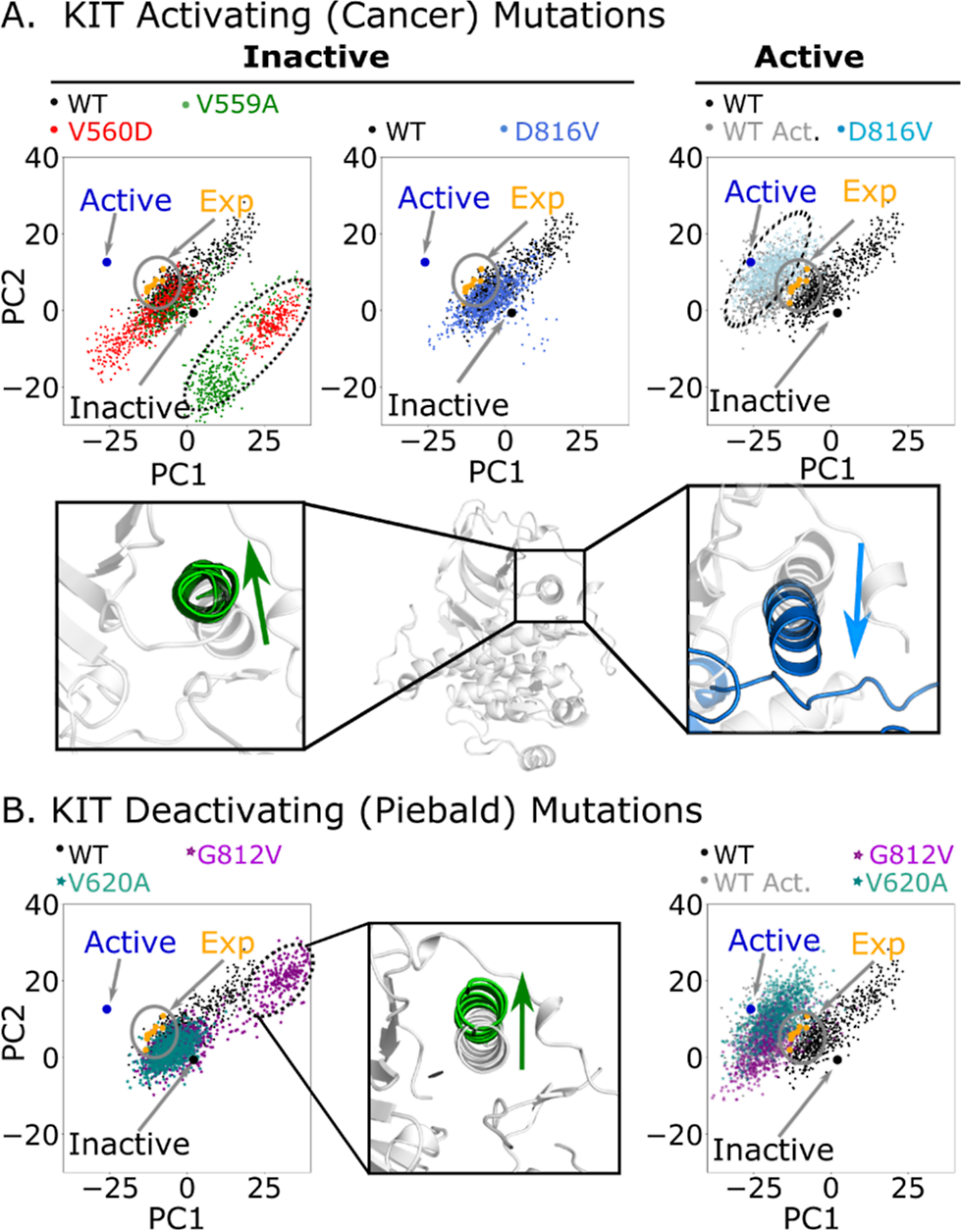
PC1 and PC2 projections of the simulated WT inactive autoinhibited KIT (black dots projected in all the panels). The experimentally determined apo inactive autoinhibited KIT (PDBid: 1t45(29)) indicated as inactive, the experimentally reported active structure (PDBid: 1pkg(4)), and other experimentally reported holo structures, including 3g0f,^23^1t46,^29^6gqk, 6gql,^34^6xv9, 6xva, and 6xvb.^35^ (A) Upper left panel presents the projections of the simulated JM mutants KIT-V559A (green dots), and KIT-V560D (red dots). The outliers (dotted circle) correspond to the movement of the αC helix deviating from the average conformation of the WT in the inactive autoinhibited form, as shown in the inset below the panel. The upper middle panel shows the projections of conformations of the simulated mutant D816V (royal blue), which overlaps with KIT-WT. The inset below presents the average structure of the KIT-WT ensemble. The upper right panel shows the projection of simulated KIT-WT (gray) and the mutant KIT-D816V (light blue) starting from the active structure. The movement associated with the new cluster of structures (dotted circle) corresponds with the downward shifting of the αC helix. (B) Right panel shows the projections of KIT-G812V and KIT-V620A, simulated from the inactive autoinhibited conformation. The inset figure shows the movement associated with the outliers enclosed in the dotted circle. The left panel shows the projection of simulated KIT-G812V and KIT-V620A starting from the active structure.

The effect of the selected mutations on the global conformational dynamics of KIT was evaluated using MD. We initially hypothesized that activating (oncogenic) mutations would shift the conformational ensemble for the inactive state toward the active state and that inactivating (piebaldism) mutations would behave oppositely, moving the conformational ensemble for the active state toward the inactive state. This hypothesis was not supported by our results (Figure 1). To represent the conformational ensembles in two dimensions, we used principal component analysis (PCA) on the WT protein in the inactive state. The first principal component (PC) involves global motions of the kinase domain, which can be roughly described as opening and closing motions of the N- and C-terminal subdomains relative to each other. The motions associated with the second PC are more focused on the activation loop and certain portions of the JM domain, as presented in Figure S2. These two first PCs account for 25.9% of the total variance of the backbone movements of the WT protein. The two mutations in the JM domain, V559A and V560D, resulted in a set of conformations differing from the main group in the simulations of autoinhibited KIT, as highlighted in the dotted circle in Figure 1A (left panel). This group of conformations corresponds to the upward shifting of the αC helix shown in the inset in Figure 1A. The inactivating mutation KIT-G812V exhibits a similar movement of the αC helix. In Figure 1B (right panel), a set of outliers highlighted in a dotted circle correspond with the upward movement of the αC helix, as presented in the inset of Figure 1B. The ensemble of conformations of the inactive autoinhibited oncogenic mutant KIT-D816V and the piebald mutant KIT-V620A overlaps with the conformational space explored by KIT-WT, but the conformational space of the mutants is apparently reduced, as presented in Figure 1A,B (left panels). The PCA projections of simulated conformations starting from the active state of the KIT protein showed a downward movement of the αC helix (Figure 1A,B), which opposes the direction of the movement induced by the mutations V559A, V560D, and G812V in the inactive conformation. We did not perform PCA analysis on the JM domain mutations using the active KIT structure because the active structure does not include the JM domain, as shown in Figure S1. Experimentally reported structures were also projected into the PCA space. Both of the starting conformations, namely, the active and inactive autoinhibited conformation, overlap with the ensemble of conformations for the active and inactive autoinhibited states, respectively. The other experimentally determined structures overlap with the ensemble of conformations explored by the KIT-WT starting from the inactive state (Figure 1, orange dots in the gray circle).

We also employed other metrics to assess whether mutations drove changes in protein conformational dynamics commonly associated with protein activation, including changes in the catalytic-spine and regulatory-spine alignment, DFG motif orientation, distances among catalytic side chains of K623, E640, and D810 distance, and the HDR motif orientation. (For explanations about the association of these metrics with kinase activation, see Figures S4–S7). In some cases, the activating (oncogenic) mutations induced conformational changes that are qualitatively consistent with a shift toward the active state. For example, introducing the D816V mutation in the inactive kinase structure resulted in conformational changes in the spine residues (toward positive values of PC1 in Figure S5) partially consistent with kinase activation. Similarly, the DFG motif sampled ”in” (active) conformations for D816V, even when starting from the inactive kinase structure, with the DFG motif in the ”out” conformation (Figure S6). However, we observed no significant differences in the catalytic side chain distances (Figure S7) or the HDR orientation (Figure S4) for D816V, and the changes induced by the other activating mutations, V559A and V560D, were generally more subtle. None of the mutations, either activating or inactivating, induced substantial changes in the conformational ensemble when starting from the active state. The inactivating mutations did result in subtle changes in the spine residues that are clearly distinct from those induced by the inactivating mutations but otherwise showed insignificant effects on the catalytic side chain distances, HDR motif orientation, and DFG in/out. In summary, these metrics do not allow us to clearly differentiate oncogenic from piebald mutations, when applied to our set of unbiased molecular dynamic simulations.

### 2.2 Oncogenic Mutations V559A, V560D, and D816V Destabilize the Inactive Autoinhibited Conformation of KIT

**Figure 2:**
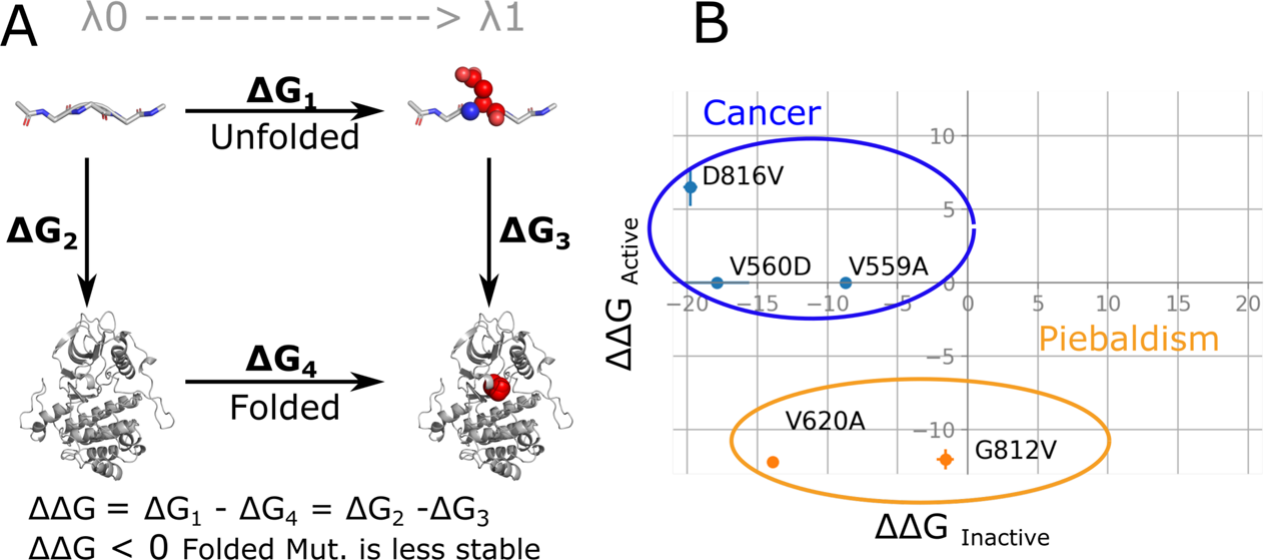
(A) Thermodynamic cycle of folding KIT-WT and mutant. The mutation site is indicated by red spheres for visualization purposes. (A) Unfolded protein is represented with the tripeptide GXG (X = V, A, or D), and the folded protein can correspond to either the inactive or active conformation of KIT. (B) 2D plot of the ΔΔ*G* of folding active and inactive mutants of KIT. The combination of destabilizing the inactive state and overstabilizing the active state is found in cancer-causing mutations, which is represented here in blue dots, while destabilization of the active conformation is found in piebaldism mutations, represented in orange.

**Figure 3:**
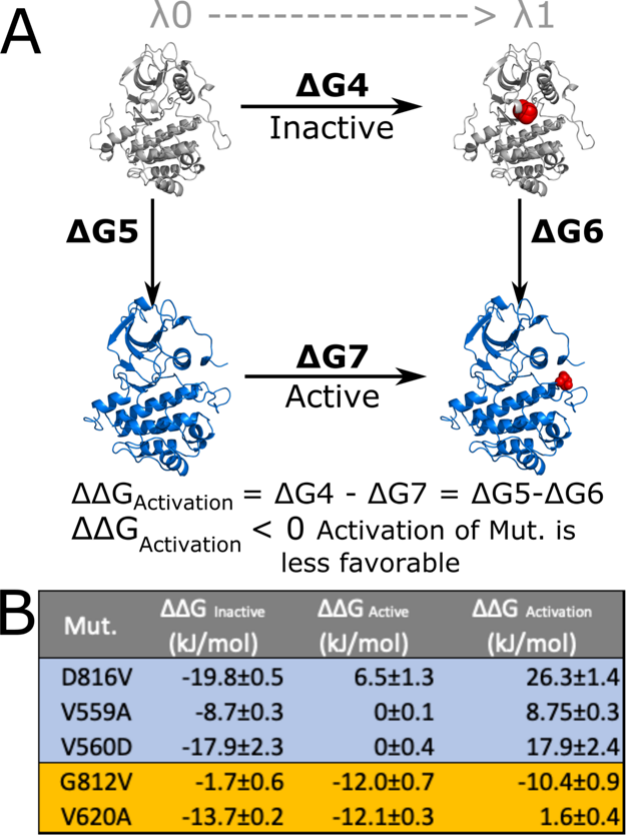
(A) Thermodynamic cycle of activating the KIT-WT and mutants. The mutation site is indicated by red spheres for visualization purposes. The inactive, autoinhibited form of KIT is presented here in gray, and its active form is presented in blue. The mutation shown here is located in the activation loop. (B) Summary of ΔΔ*G*_Inactive_, ΔΔ*G*_Active_, and ΔΔ*G*_Activation_. Values for Δ*G*_1_, Δ*G*_4_, and, Δ*G*_7_ are provided in Figure S3. Oncogenic mutations are highlighted in blue, and piebald mutations are highlighted in orange.

We tested our hypothesis that oncogenic mutations would thermodynamically destabilize the autoinhibited state of KIT using alchemical free energy calculations. Specifically, we evaluated the relative free energy of WT and mutant proteins (see Figure S1) for the folded autoinhibited state relative to the unfolded protein, represented by a tripeptide GXG (X = A, V, or D). As shown in the thermodynamic cycle of Figure 2A, the free energy difference between folding the KIT-WT (Δ*G*_2_) and mutant (Δ*G*_3_) is equal to the free energy difference between alchemically changing the WT to mutant in the unfolded (Δ*G*_1_) and folded (Δ*G*_4_) states. Consequently, if the calculated ΔΔ*G*_Inactive_ < 0, then the mutant is thermodynamically less stable than the WT, which is the case for all three mutations D816V, V559A, and V560D, as summarized in Figures 2B and 3B. The negative ΔΔ*G*_Inactive_ for the activation loop and the JM domain mutations are consistent with these mutations destabilizing the inactive conformation.

### 2.3 Oncogenic Mutation D816V Additionally Overstabilizes the Active Conformation

The transition from the autoinhibited to the active state involves several conformational changes, including changes in the JM domain and activation loop. We hypothesized that mutations may differentially impact the stability of the autoinhibited and active states. We carried out additional alchemical free energy simulations to assess the effect of the D816V oncogenic mutation on the active state of KIT. We did not attempt to perform analogous calculations on the JM domain mutations because the structure of active KIT does not include the JM domain, which is presumed to be unstructured in the active state or perhaps interacting with the cell membrane. The same thermodynamic cycle of Figure 2A can be applied to the active state for D816V, which is predicted to thermodynamically stabilize the active state relative to WT ΔΔ*G*_Active_ = 6.5 ± 1.3 kJ/mol (Figures 2B and 3B). Both the destabilization of the autoinhibited state and the stabilization of the active state could in principle contribute to increased activity of KIT-D816V. Using the thermodynamic cycle in Figure 3A, we can compare the free energy of transition from the autoinhibited to the active state between the WT (Δ*G*_5_) and mutant (Δ*G*_6_), which is equivalent to the free energy difference of mutating the WT to the mutant in their corresponding inactive (Δ*G*_4_) or active (Δ*G*_7_) folded state. Here, the positive ΔΔ*G*_Activation_ = 26.3 ± 1.4 kJ/mol indicates a more favorable activation of the mutant D816V in comparison to the KIT-WT. The combined results point to a double effect of the activation loop mutation D816V, both destabilizing the inactive conformation and overstabilizing the active conformation, as presented in Figure 2B.

### 2.4 Piebald Mutations Destabilize the Active State

We hypothesized that piebald loss-of-function mutations (see Figure S1) should, in essence, behave oppositely to the oncogenic mutations, destabilizing the active state rather than the inactive state. We tested our hypothesis following the same thermodynamic cycle in Figure 2A. We evaluated the free energy of the WT and mutant for the folded active state relative to the unfolded protein, represented by a tripeptide GXG (X = G, V, or A). The calculated ΔΔ*G*_Active_ < 0 entails a thermodynamically less stable mutant than the WT, for both piebald mutations G812V and V620A, as summarized in Figures 2B and 3B. These negative ΔΔ*G*_Active_ are consistent with the mutations destabilizing the active conformation.

The evaluation of the free energy of the folded inactive state resulted in a calculated ΔΔ*G*_Inactive_ = −1.6 ± 0.5 for the G812V mutation located in the DFG motif of the activation loop. We could interpret that this mutation has a marginal effect on the overall stability of the inactive autoinhibited KIT conformation, according to errors observed typically on computational free energy calculation compared to experiments.^36,37^ The calculated ΔΔ*G*_Inactive_ = −13.7 ± 0.2 for the KIT-V620A mutation predicts the destabilization of the inactive conformation, as can be summarized in Figures 2B and 3B.

## 3 Discussion

Through the use of MD simulations and MD-based free energy calculations, we assessed how KIT mutations affect the dynamics and thermodynamic stability of KIT. Our results predict that oncogenic mutations in the JM domain induce a movement in the αC helix that is opposite to the commonly reported movement of this helix upon kinase activation (Figure 1A). We hypothesize that this effect may alter the kinase domain dynamics independent of other reported environmental or external factors such as phosphorylation^38^ or protein–lipid interactions.^39^ In addition, the predicted conformational change resembles the conformational change induced by the JM-mutation Y610F in the tyrosine kinase ephrin type-B receptor 2 (EPHB2).^40^ Interestingly, such conformational rearrangement was not observed for the D816V mutation. We hypothesize that the upward positioning of the αC helix could be a common feature among oncogenic JM-mutations in the tyrosine kinase family but not so for the activation loop mutants. The piebald mutation G812V induced an upward movement of the αC helix similar to that observed for the JM domain mutations V559A and V560D. The G812V mutation substitutes the Gly in the DFG motif for Val and may affect the DFG motif orientation, an essential component of activity regulation in type III receptor tyrosine kinases.^19,41^ We hypothesize that the relocation of the αC helix induced by the oncogenic JM mutations and the piebald G812V mutation reflects the complex interaction between the activation loop, the JM domain, and αC helix, tightly regulating activation of KIT kinase, and may have implications for drug binding. Other parameters to assess functionally relevant conformational changes—such as the catalytic- and regulatory-spine alignment, DFG motif orientation, and distances among the catalytic residues—did not, in general, provide useful discrimination among activating and deactivating mutations.

Beyond the dynamics and structural changes induced by the oncogenic JM domain mutations V559A and V560D in KIT and the piebald mutation G812V, the thermodynamic stability of the mutant *versus* KIT-WT provides insights into how all the mutations, in spite of their effect on the protein dynamics, can lead to the activation or deactivation of the KIT receptor. Here, we propose that the transition from the inactive to the active conformation of KIT is the sum of two contributions: (1) the stabilization/destabilization of the autoinhibited inactive conformation and (2) the stabilization/destabilization of the active conformation, as represented in Figure 3A.

All the studied oncogenic mutations have the potential to destabilize the autoinhibited inactive form, as summarized in Figures 2B and 3B. We speculate that the additional contribution, which magnifies the effect of an oncogenic mutation, comes from the effect of the active form of KIT. Once the kinase is in the active state, we hypothesized that the JM domain will likely be exposed to the solvent as an unfolded domain, which will result in an additional ΔΔ*G*_FoldActive_ ≈ 0. As a consequence, only the activation loop mutations D816V will have a contribution in the stability of the active form of the KIT protein. Our calculation resulted in a ΔΔ*G* = 6.5 ± 1.3 kJ/mol and shifting the overall ΔΔ*G* of activation from 19.8 ± 0.5 to 26.3 ± 1.4 kJ/mol, which suggests an overstabilization of the activated form of D816V KIT. Thus, if we consider that the ΔΔ*G* of activation encompasses both contributions, the inactive and active state, as presented in Figure 3A, the D816V mutation in the activation loop will dramatically shift the conformation of KIT to its active form in comparison to the mutations in the JM domain.

The piebald mutations have the potential to destabilize the active form of the KIT kinase, as summarized in Figures 2B and 3B. We hypothesized that additional contributions to hinder the activation of the KIT kinase come from further destabilizing the inactive autoinhibited state or overstabilizing the inactive state. The mutation G812V is an example of destabilization of the active state as the main piebald disease driver presented in Figures 2B and 3B. The ΔΔ*G* = −1.6 ± 0.4 represents a marginal effect of G812V mutation on the inactive autoinhibited KIT, compared to the biggest contribution of ΔΔ*G* = −12.0 ± 0.7 of active KIT. There is overall destabilization of the KIT kinase domain with the V620A mutation, expressed as negative ΔΔ*G* for the inactive and active states simultaneously presented in Figure 2B. The destabilization of both states is similar, with ΔΔ*G* = −13.7 ± 0.2 and ΔΔ*G* = −12.1 ± 0.3 for inactive and active states, respectively. These results highlight the importance of each ΔΔ*G* contribution individually, instead of a total ΔΔ*G* for activation, which, in the case of mutant V620A, amounts to 1.6 ± 0.4 (Figure 2B) and may lead to the misinterpretation of a marginal effect over the protein activation.

The prevalence of the active versus the inactive state of KIT dictates how well different tyrosine kinase inhibitors bind and inhibit the protein. First generation KIT inhibitors are ineffective against mutations in the activation loop such as D816V.^42,43^ In contrast, newer generation inhibitors, such as midostaurin and avapritinib, are effective against D816V.^25,26^ Therefore, mutants that stabilize the active conformation are expected to be resistant to first generation inhibitors but sensitive to new generation inhibitors. We speculate that MD-based free energy calculations could predict the shift toward the active form of the kinase by a mutation, thereby providing insights into which inhibitors would be effective. For example, if MD predicts that a mutant stabilizes the active conformation, newer generation inhibitors should be first line therapy since older drugs (imatinib, sunitinib, and regorafenib) will likely be ineffective.

The ability to use our methodology to predict the impact of mutations has important implications for KIT-targeted therapy. Genetic profiling of tumors has become commonplace; however, the most frequent mutations detected are variants of unknown significance (VUS).^44^ Often these variants are in druggable proteins, such as KIT. This creates a challenge for clinicians who are faced with the questions of (1) whether targeted therapy is indicated and/or safe for their patients and (2) which targeted therapy to prescribe.^45,46^ The results presented in this manuscript provide an important step forward for defining the functional significance of KIT VUSs: the MD-based free energy calculations presented here could be used to predict both the functional significance and the most appropriate therapy for patients with KIT VUSs.

The conformational specificity of kinase inhibitors is not unique to KIT. PDGFR, FLT3, and other type 3 receptor tyrosine kinases share the same basic domain structure as KIT, including an extracellular ligand binding domain, an autoinhibitory JM domain, and an intracellular kinase domain.^1^ PDGFR and FLT3 have recurrent oncogenic mutations in the activation loop that are analogous to KIT-D816V (PDGFR D842V and FLT3 D835Y) and activating mutations in the JM domain (*e.g.*, FLT3 internal tandem duplications).^19^ PDGFR and FLT3 also have approved kinase inhibitors that are mutation-specific and thus conformation-specific. For example, imatinib inhibits inactive PDGFR and avapritinib inhibits active PDGFR.^47^ For FLT3, sorafenib inhibits the inactive conformation, whereas gilteritinib is a potent and specific inhibitor of the active conformation.^48^ By generating similar thermodynamic cycles and free energy calculations for other oncogenic kinases similar to KIT, such as FLT3 and PDGFR, we may be able to generate a powerful computational platform to predict the functional significance and druggability of VUS in these proteins. Such a platform would advance individualized medicine and expand the number of patients who can benefit from tumor sequencing.

## 4 Materials and Methods

### 4.1 Molecular Dynamics

#### 4.1.1 Starting Conformations

Starting conformations of apo structures of the KIT kinase in the inactive, autoinhibited conformation, which includes the JM domain, and in the active conformation were taken from X-ray crystallographic structures (PDB ID codes: 1T45(29) and 1PKG,^4^ respectively). Three oncogenic mutations V559A, V560D, and D816V and two piebald mutations G812V and V620A were introduced in the inactive conformation. The oncogenic mutation D816V mutation and both piebald mutations G812V and V620A were introduced in the active form, using the package Pymol.^49^ Structures of the tripeptides GXG (X = Ala, Val, Gly, or Asp) were obtained from the pmx server.^50^

#### 4.1.2 Equilibrium Molecular Dynamics Simulations

All simulations were performed using the package GROMACS, version 2020.1.^51^ The following combination of force fields was chosen: Amber14SB^52^ was used for the protein, Joung ion parameters^53^ were chosen for the ions, and the TIP3P model^54^ was chosen for the water molecules. The potential energy of the protein was minimized during 50 000 steps in vacuum using the steepest descent algorithm.^55^ Then, ≈13 630 to 16 870 water molecules, ions at a concentration of 0.15 M, and counterions to neutralize the system were added, in a rectangular box, of unit cell dimensions ≈8–9 × 7.5 × 8.5 nm^3^, for a total of ≈436 000 atoms. The solvated system was energy-minimized for 50 000 steps (also with the steepest descent algorithm). Three replicas were generated from this minimized system. The solvent was equilibrated in each replica during 10 ns, by maintaining the protein position restrained with harmonic springs on the heavy atoms (elastic harmonic constant of 1000 kJ/mol/nm^2^). The position restraints were removed, and production runs of 500 ns per replica were performed. Overall, the cumulative simulation time was ≈9 μs.

All simulations were performed in the isothermal–isobaric (*NPT*) ensemble, coupling the system to the Berendsen barostat^56^ at a reference pressure of 1 bar and a coupling constant of 1 ps. The temperature was kept constant at 310 K, separately for the protein and for the water and ions, using the Nosé–Hoover thermostat^57,58^ (time constant of 0.5 ps). The center of mass of the system was removed every 100 steps. The long range electrostatic interactions were treated with the particle mesh Ewald technique,^59,60^ in the real space for distances below 1.0 nm and in the reciprocal space beyond these distances. Short-range interactions were considered through a Lennard-Jones potential within a cutoff distance of 1 nm. Nonbonded neighbors were assigned through the Verlet buffer scheme.^61^ Bonds involving hydrogen atoms were constrained using the LINCS algorithm,^62^ and bonds and angles of water molecules were treated with SETTLE,^63^ hence allowing the integration of equations of motion at discrete time steps of 2 fs.

### 4.2 Free Energy Calculations

MD-based alchemical free energy calculations were used to compute the relative thermodynamic stabilization of the kinase domain in either the active or inactive state of the various point mutants (V559A, V560D, D816V, G812V, and V620A) following the thermodynamic cycle in Figure 2A.

Using the alchemical transformation from the WT (λ0) to mutant (λ1), we calculated the work needed to transform the system from one state to another through a nonphysical pathway.^37^ In these transitions, λ couples the Hamiltonians of the states as follows: *H* = (1 – λ)·*H*_WT_ + λ·*H*_MUT_. The work associated to a single transition is computed by . Subsequently, the work distribution associated to “forward” [*P*_f_(*W*)] and “reverse” [*P*_r_(*W*)] transitions enables the calculation of the free energy difference (Δ*G*), using the Crooks fluctuation theorem^64^ described by , where β = 1/(*k*_B_*T*), with *k*_B_ being the Boltzmann constant and *T* being the temperature. Finally, the Bennett acceptance ratio (BAR) as a maximum likelihood estimator, proposed by Shirts *et al.*,^65^ was used to derive the equilibrium free energy (Δ*G*) from the distribution of the nonequilibrium simulations. Assuming equal number of forward and reverse transitions, the BAR maximum likelihood is expressed as follows: . Finally, the Δ*G* uncertainty was calculated by bootstrapping, solving the last equation 100 times from randomly selected forward and reverse data sets.

Four hundred and fifty conformations of folded inactive and active proteins, obtained from the MD equilibrium simulations, served as starting structures. The hybrid topologies needed for alchemical calculations were obtained with the *pmx* tool.^37^ All the starting conformations were minimized for 100 000 steps and further equilibrated for 500 ps, previous to the forward or backward transformations of 100 ps each. Similarly, three replicas of the tripeptides GXG (X can be replaced by Val or Asp), representing the unfolded KIT protein, were simulated in unbiased MD-simulation. Four hundred and fifty conformations were taken as starting points for the forward and backward calculations, following the previously described protocol. All the simulations were performed with the GROMACS 4.6 version with an integrated soft-core potential function^66^ using all the previously described parameters. The final free energies and their associated uncertainty were estimated using the BAR method^67^ implemented in the *pmx* set of tools.

### 4.3 Simulation Analysis

The PCA was performed using the tools *covar* and *anaeig* in GROMACS 2020.1. The combined trajectories of the backbone residues 571–688 and 763–930 of the three replicas of the WT protein starting from the autoinhibited-inactive form functioned as a reference to construct the covariance matrix assessing the global motions of the protein. All the trajectories, including the JM mutations, the activation loop mutations, the piebald mutations, and the WT trajectories starting from the active conformations, were projected into this reference PCA construct. Additionally, the reported structures in the protein data bank (PDB), 1t45 (inactive conformation),^29^1pkg (active conformation),^4^3g0f,^23^1t46,^29^6gqk, 6gql,^34^6xv9, 6xva and 6xvb,^35^ were also mapped into the PCA matrix.

We measured features associated with kinase activation, such as the catalytic- and regulatory-spine alignment,^68^ DFG motif orientation (in/out),^69^ HDR motif orientation,^70^ and distances between catalytic side chains, K623–E640 and K623–D810.^30^ The catalytic- and regulatory-spine alignment was assessed applying PCA to residues A621, V603, L678, L798, L7999, and L800 of the catalytic-spine and residues L656, L644, F811, and H790 of the regulatory-spine, following the protocol previously described. DFG motif orientation was monitored by measuring Φ (atoms C–N–CA–C) and Ψ angles (atoms N–CA–C–O) of residues D810 and F811. HDR motif orientation was determined by the Φ angle (atoms C–N–CA–C) of residue R791. The K623–E640 and K623–D810 distances were measured between the amine nitrogens in K623 and carboxylate oxygens in the E640 and D810 side chains. Distributions of each of these metrics were obtained from 750 evenly spaced snapshots from the unconstrained simulations.

The free energy differences were calculated following the procedure described in the Materials and Methods section for Free Energy Calculations.

## Supporting Information Available

Critical motifs in KIT activation and summary of location of activating and deactivating mutations; first two PCs of the conformational dynamics observed by MD simulations of the autoinhibited inactive WT KIT; nonequilibrium work recovered from thermodynamic transformation of the tripeptide GXG, inactive autoinhibited, and active KIT kinase; HDR motif orientation; catalytic (C)- and regulatory (R)-spine alignment; DFG-in and DFG-out orientations; distance between catalytic residues K623, E640, and D810 (PDF)
Input files used on equilibrium and Free Energy MD-simulations (ZIP)
