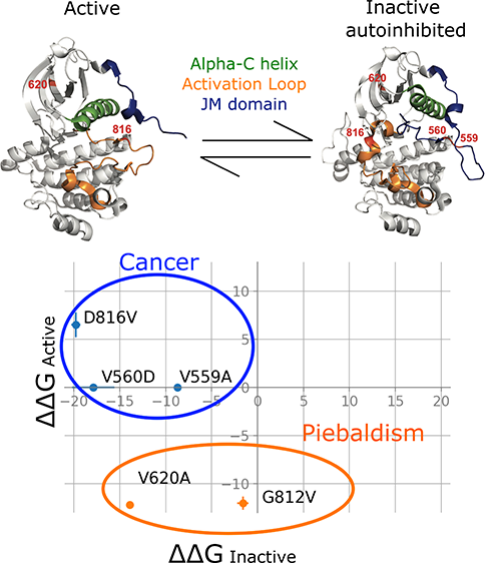# Correction to “Assessing the Activation of Tyrosine Kinase KIT through Free Energy Calculations”

**DOI:** 10.1021/acs.jctc.2c01157

**Published:** 2022-12-09

**Authors:** Angélica Sandoval-Pérez, Beth Apsel Winger, Matthew P Jacobson

In our original article (https://pubs.acs.org/doi/10.1021/acs.jctc.2c00526), we identified in the abstract figure the mislabeling of the active and inactive autoinhibited structures for the KIT kinase.

The new figure here has the correct labels for the active and inactive autoinhibited KIT kinase protein used in our simulations. The mislabeling of the structures does not affect any of the reported results.