# Supplementary material for: Assessing the Activation of Tyrosine Kinase KIT through Free Energy Calculations
Source: J Chem Theory Comput. 2022 Sep 27;18(10):6251–8. doi: 10.1021/acs.jctc.2c00526 (PMC9558371; doi:10.1021/acs.jctc.2c00526)
Supplement: Supplementary file 1 — ct2c00526_si_001.pdf [file ct2c00526_si_001.pdf]

Supplementary Material: Assessing the activation of tyrosine-kinase KIT through free energy calculations

Angélica Sandoval-Pérez[1], Beth Apsel Winger[2], and Matthew P Jacobson *[1]

[1]Department of Pharmaceutical Chemistry, University of California, San Francisco, CA, United States

[2]Department of Pediatrics, Division of Hematology and Oncology, University of California, San Francisco, San Francisco, CA, United States

*Corresponding author: matt.jacobson@ucsf.edu

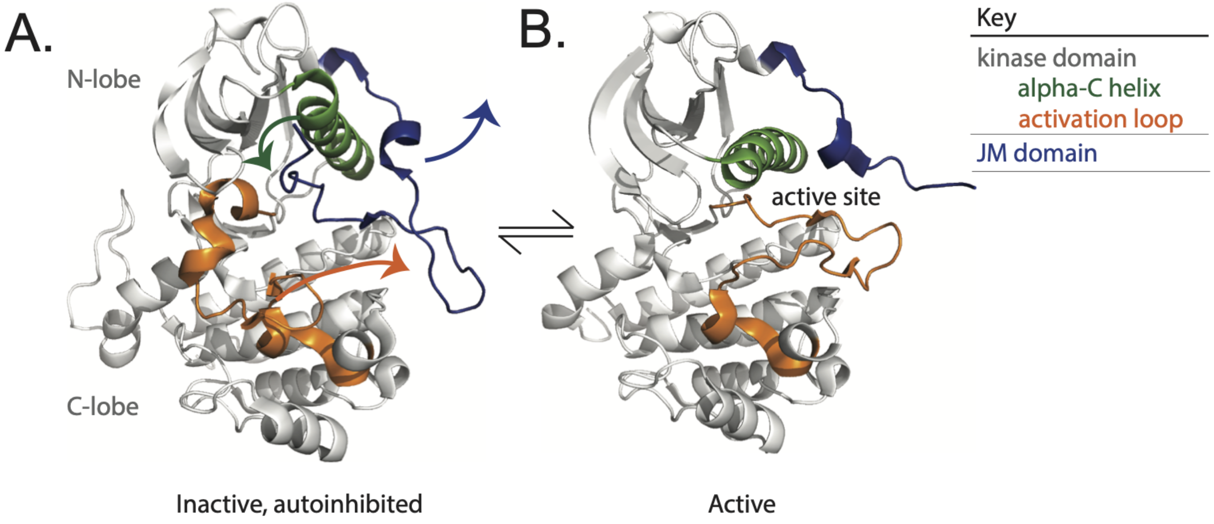

C.

Activating/ Oncogenic	De-activating/ Piebald
V559A<br>V560D<br>D816V	V620A<br>G812V

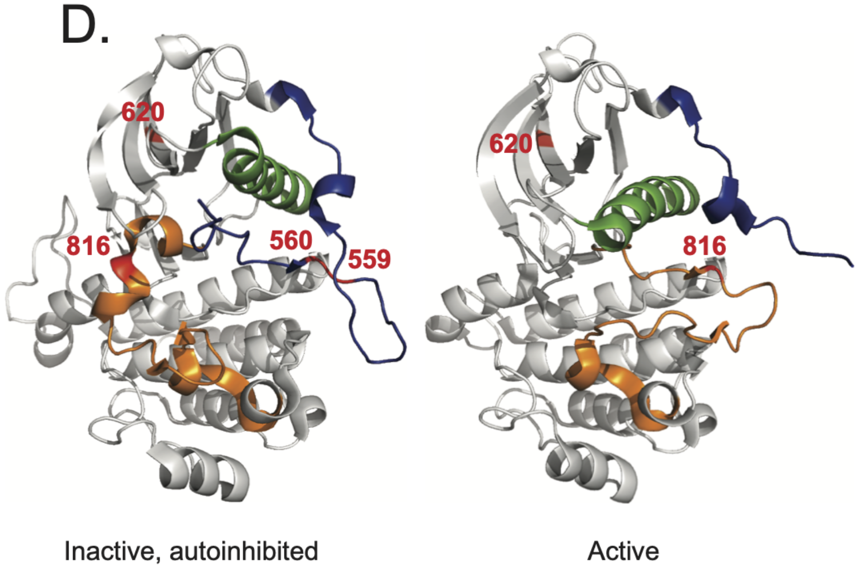

Figure S1: **Critical motifs in KIT activation and summary of location of activating and deactivating mutations.** The inactive autoinhibited structure is shown in (A) (PDB 1T45). In this structure the JM domain (blue) blocks the active site, the activation loop (orange) is folded in on the active site and alpha-C helix (green) is in a higher position within the N-lobe of the kinase. The structure of the active kinase is shown in (B). KIT activation leads to movement of the JM domain (blue) away from the active site, extension of the activation loop (orange) to a position where it can bind substrate and movement of the alpha-C helix (green) down where it can aid in catalysis. The arrows in panel A highlight the conformational changes that occur with KIT activation. A summary table classifying mutants as activating and oncogenic or deactivating and associated with Piebaldism is shown in (C) with the location of mutations (shown in red) on the structure of inactive, autoinhibited KIT and active KIT shown in (D).

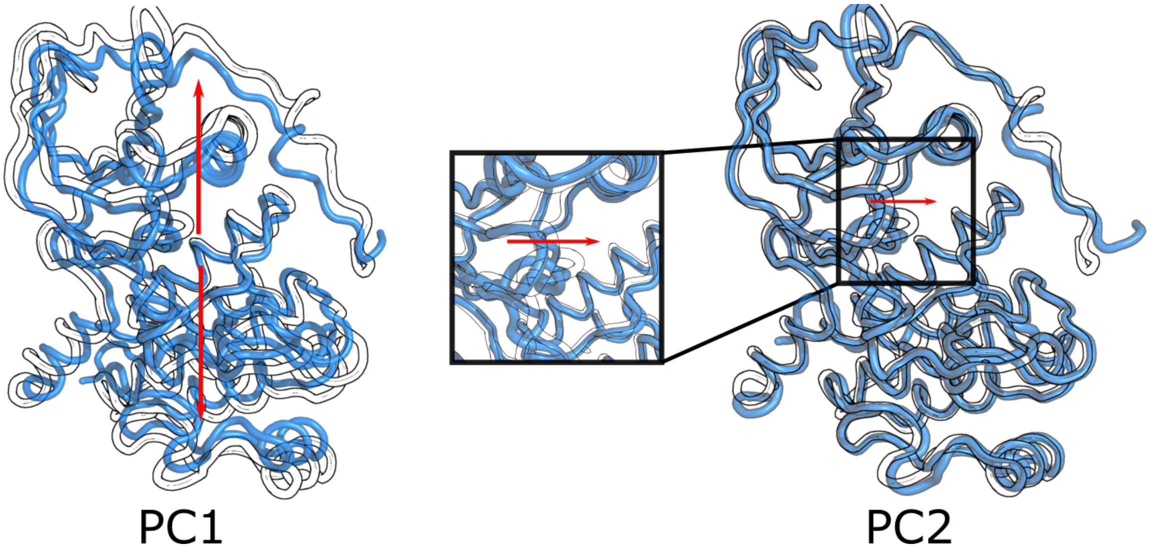

Figure S2: **First two principal components of the conformational dynamics observed by molecular dynamics simulations of the auto-inhibited-inactive wild type KIT.**

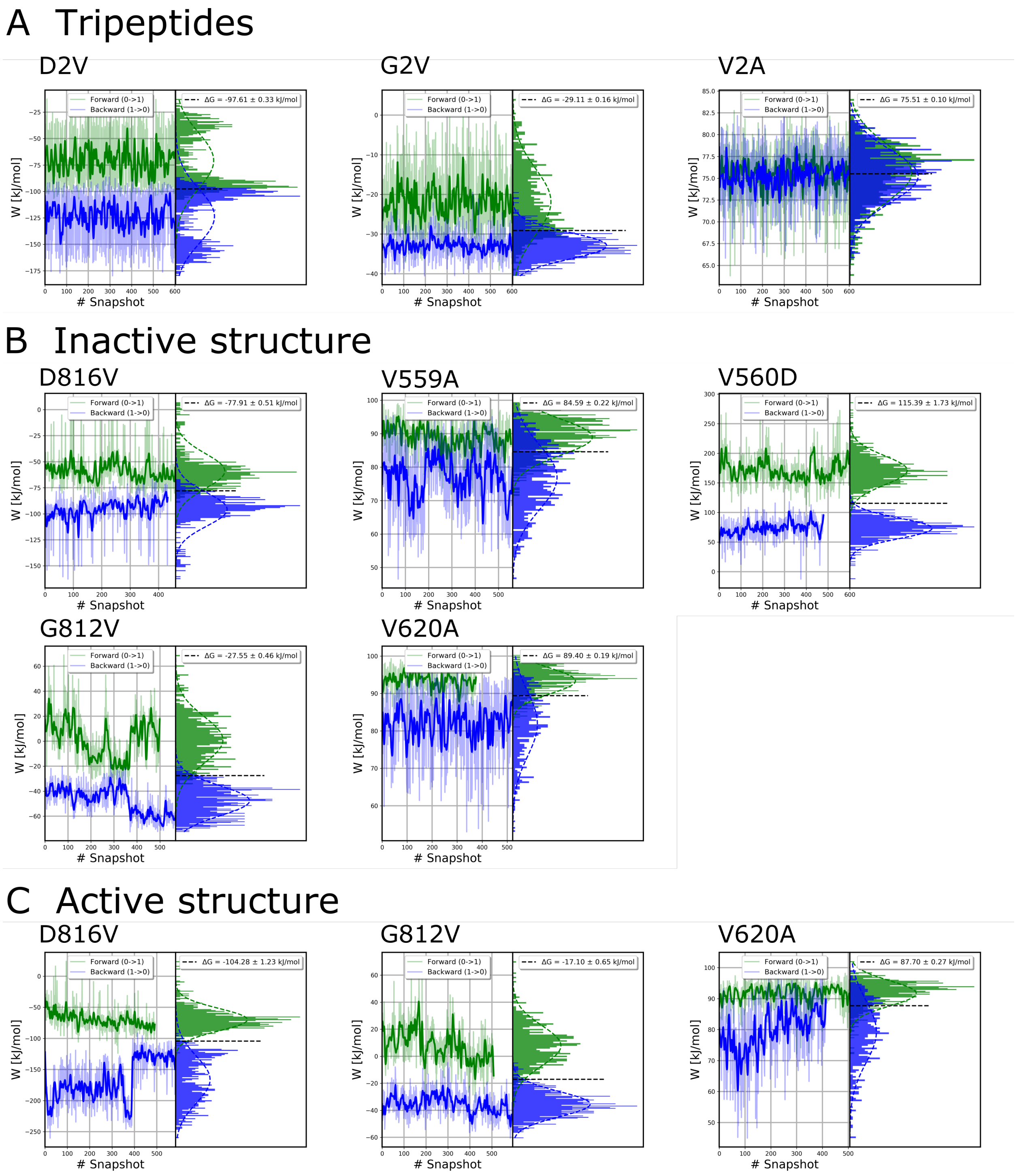

Figure S3: **Non-equilibrium Work** recovered from thermodynamic transformation of the tripeptide GXG (A), Inactive-autoinhibited (B), and active KIT kinase (C).

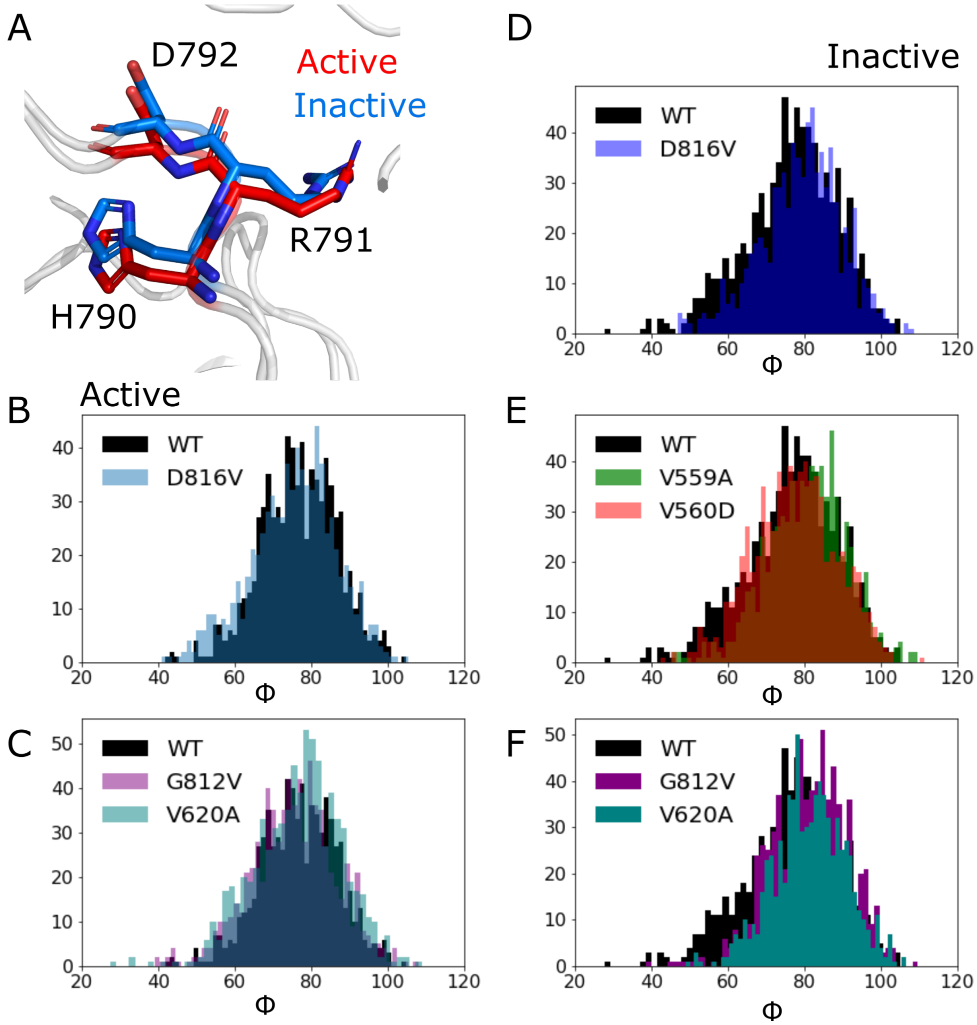

Figure S4: **HDR motif orientation.** The HDR motif is a series of three amino acids (His, Asp, Arg) located close to the active site that forms hydrogen bonding networks that are thought to play an important role in kinase activation.[?] The active conformation of the kinase will stabilize the backbone torsional angle ϕ of R791 (atoms C-N-CA-C) between 40° and 100° (a value of 59.01° was reported by La et al.[?]), while the inactive conformation should allow the backbone flexibility shifting the ϕ angle towards negative values (Panel A). Nevertheless in our set of simulations, no significant differences were observed between active and inactive autoinhibited conformations, wt or mutants (Panels B-F).

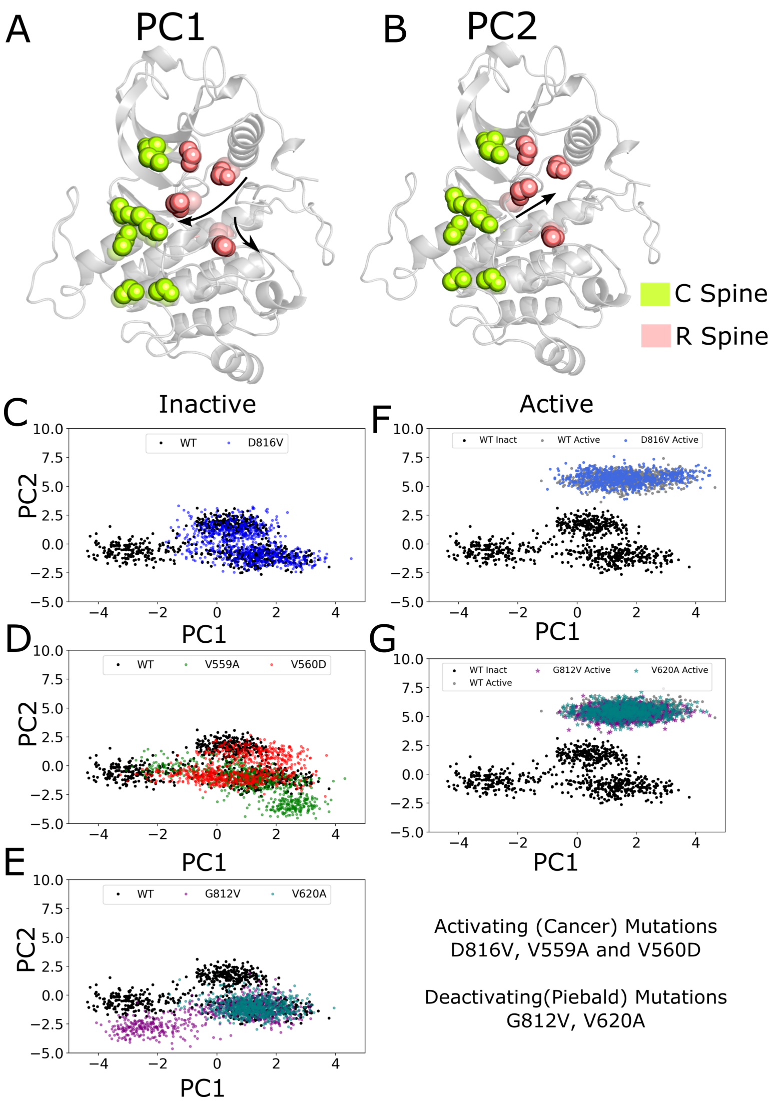

Figure S5: **Catalytic (C)-and Regulatory (R)-Spine alignment.** The inactive KIT structure with the residues of the C-spine and the R-spine highlighted. These spines are composed of series of non-contiguous amino acids (panel A and B) that spatially align once the kinase is activated. The C-Spine was defined by the backbone of residues A621, V603, L678, L798, L7999, L800, and the R-Spine included the backbone of residues L656, L644, F811, and H790. R-Spine alignment has been reported as a hallmark of an active kinase. Our PCA analysis demonstrates the largest changes occur in the R-Spine as depicted by the projected movements PC1 and PC2 (panel A-B). The R-Spine alignment allow to differentiated between Inactive and Active, as presented in the 2D projections named as Active (panels F-G). Observable differences on activating and deactivating mutations are observed for ensembles simulated starting from the inactive autoinhibited conformation. Activating mutations shift the ensemble towards positive values of PC1 (panels C-D), while deactivating mutations shift the PC2 towards negative values (panel E).

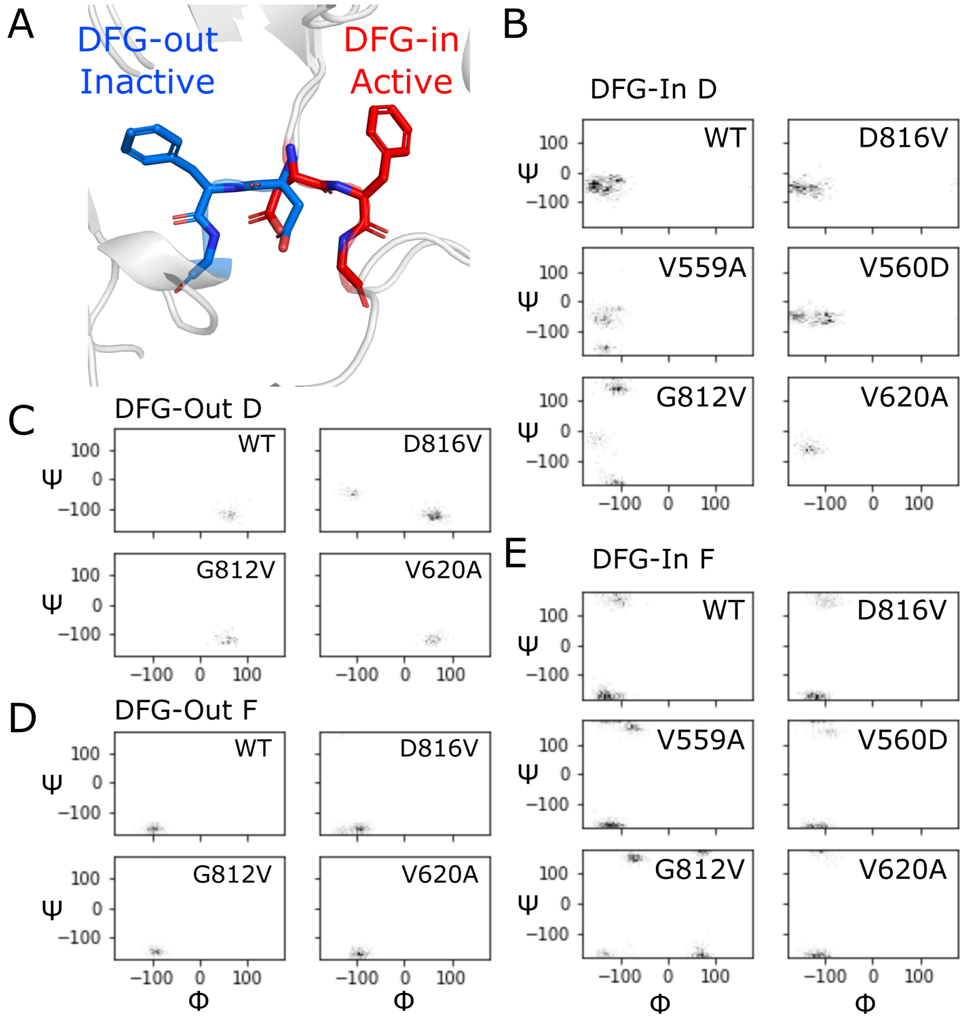

Figure S6: **DFG-in and DFG-out orientations**. The DFG motif (residues 810-812) is an absolutely conserved motif at the beginning of the activation loop whose orientation changes drastically between the active and inactive kinase (Panel A). In active conformations of KIT the F811 residues is packed in a hydrophobic pocket between the N lobe and the C lobe. Inactivation of KIT occurs when F811 moves out and sterically blocked the ATP binding site. We evaluated the orientation of the DFG motif by calculating the angle distribution of the backbone angles ϕ and ψ of residues D810 and F811. The ϕ angle was formed between the atoms C-N-CA-C and the ψ angle between the atoms N-CA-C-O. All the mutations explored conformations equivalent to the WT ϕ and ψ angles in the corresponding active (DFG-In) and Inactive auto-inhibited (DFG-Out) conformations. D816V starting from DFG-Out conformation was the only mutation showing some level of flipping between DFG-In and DFG-Out.

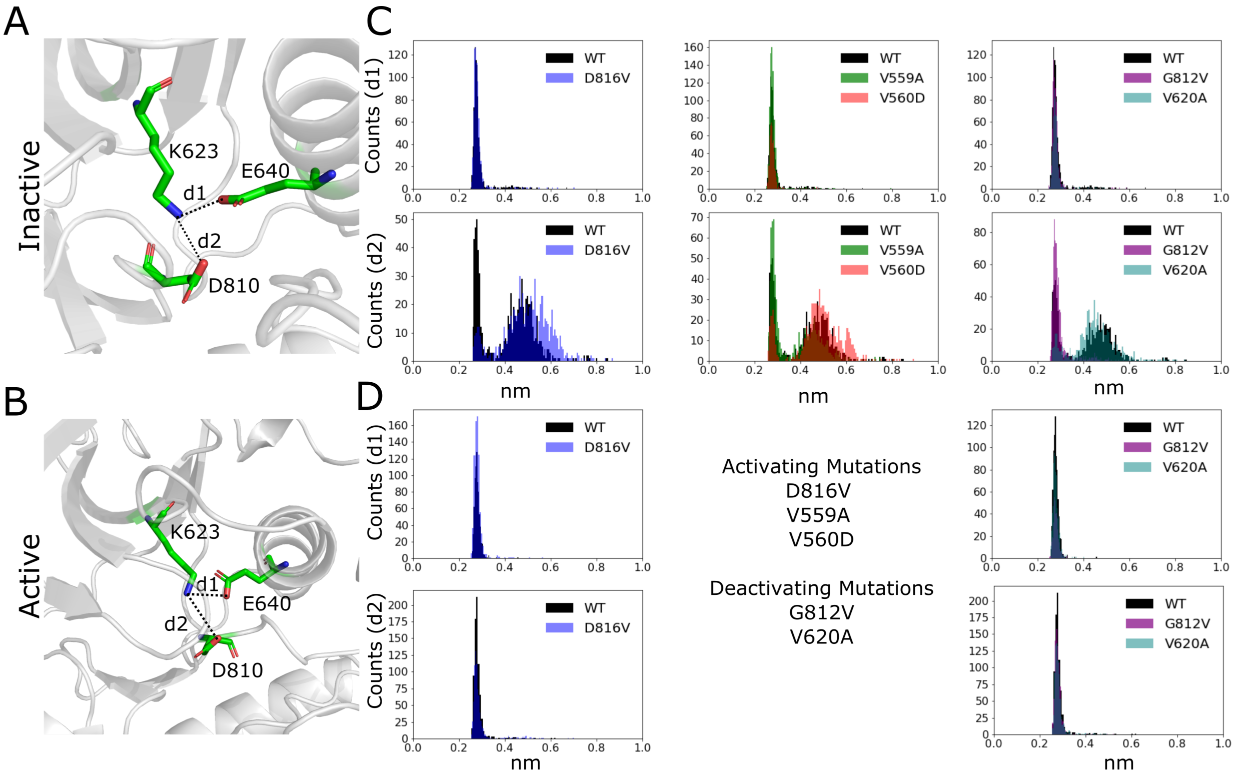

Figure S7: **Distance between catalytic residues K623, E640 and D810**. In the active kinase, K623, E640 and D810 orient in a specific way to enable phosphoryl transfer (the catalytic activity of the kinase). The distances between residues K623 and E640 (d1), and K623 and D810 (d2) help to differentiate between active and inactive conformations (panels A-B). In the active conformation the two salt bridges K623 and E640 (d1), and K623 and D810 (d2) are formed and stabilized (panel D). While in the inactive autoinhibited conformation, the salt bridge formed between K623 and D810 can break, producing a bimodal distribution as observed in the d2 of the Inactive conformation (panel C). The distances were calculated between atoms NZ in K623 and OD* in D810 and OE* in E640.

References

[1] La Sala G., Riccardi L.and Gaspari R., Cavalli A., Hantschel O., and De Vivo M. HRD motif as the central hub of the signaling network for activation loop autophosphorylation in abl kinase. *J. Chem. Theory Comput.*, 12(11):5563–5574, 2016.
